# Privacy-Aware Relevant Data Access with Semantically Enriched Search Queries for Untrusted Cloud Storage Services

**DOI:** 10.1371/journal.pone.0161440

**Published:** 2016-08-29

**Authors:** Zeeshan Pervez, Mahmood Ahmad, Asad Masood Khattak, Sungyoung Lee, Tae Choong Chung

**Affiliations:** 1 School of Engineering and Computing, University of the West of Scotland, Paisley, PA1 2BE, United Kingdom; 2 Ubiquitous Computing Lab, Department of Computer Engineering, Kyung Hee University, Global Campus, 1 Seocheon-dong, Giheung-gu, Yongin-si, Gyeonggi-do 446-701, South Korea; 3 College of Technological Innovation, Zayed University, Abu Dhabi, United Arab Emirates; 4 Artificial Intelligent Lab, Department of Computer Engineering, Kyung Hee University, Global Campus, 1 Seocheon-dong, Giheung-gu, Yongin-si, Gyeonggi-do 446-701, South Korea; Kaohsiung Medical University, TAIWAN

## Abstract

Privacy-aware search of outsourced data ensures relevant data access in the untrusted domain of a public cloud service provider. Subscriber of a public cloud storage service can determine the presence or absence of a particular keyword by submitting search query in the form of a trapdoor. However, these trapdoor-based search queries are limited in functionality and cannot be used to identify secure outsourced data which contains semantically equivalent information. In addition, trapdoor-based methodologies are confined to pre-defined trapdoors and prevent subscribers from searching outsourced data with arbitrarily defined search criteria. To solve the problem of relevant data access, we have proposed an index-based privacy-aware search methodology that ensures semantic retrieval of data from an untrusted domain. This method ensures oblivious execution of a search query and leverages authorized subscribers to model conjunctive search queries without relying on predefined trapdoors. A security analysis of our proposed methodology shows that, in a conspired attack, unauthorized subscribers and untrusted cloud service providers cannot deduce any information that can lead to the potential loss of data privacy. A computational time analysis on commodity hardware demonstrates that our proposed methodology requires moderate computational resources to model a privacy-aware search query and for its oblivious evaluation on a cloud service provider.

## Introduction

We are living through a post-PC era in which computing facilities are regarded as the fifth utility [1]. These facilities, which are primarily related to computational and storage services, are provisioned to subscribers on a pay-as-you-go basis. This new service provisioning model is known as cloud computing [2]. Advances in virtualization technologies and the availability of high-speed Internet have fostered this on-demand computing paradigm. It provides an abstraction of unlimited computational and storage facilities to its subscribers, enabling them to dynamically scale services or applications according to their specific requirements [3]. These on-demand and virtualized services are provisioned by a cloud service provider (CSP). The underlying cloud infrastructure (processing power, storage capacity, and networking facility) is owned, managed, and operated by a CSP. Subscribers do not need to take care of the cloud infrastructure, the assurance related to uninterrupted service provisioning is delineated in a service contract that is signed between the CSP and its subscribers.

Cloud-based storage service is a generalization of cloud-enabled data sharing, archiving, collaboration, and synchronization services [4]. These services leverage their subscribers to store their data for much a longer duration without the concerns of data availability and accessibility from varied devices, i.e., desktop computers, laptops, and smartphones. As lucrative as it sounds, there are data privacy concerns when confidential and personal data are outsourced to cloud-based storage services owned and managed by a CSP [5], [6], [7], [8]. Since these services are provisioned beyond the federated domain of subscribers over which they do not have any control, the CSP is considered to be an untrusted entity [9]. The most obvious solution to ensure data confidentiality in untrusted domain is to encrypt personal and confidential data before it can be outsourced to a cloud-based storage service. Since these services are provisioned on a pay-as-you-go basis, each data access request is charged according to the amount of data transferred between the subscriber and the CSP. Thus, the capability of a subscriber to access relevant encrypted data is very important. It ensures data privacy and can also increase the utility of the cloud-based storage services.

To access relevant data within the untrusted domain of a CSP, two main methodologies are employed, namely a search over encrypted data [10], [11], [12] and an index-based data search [13], [14], [15]. A search over encrypted data exploits the mathematical properties (trapdoors) of the cryptographic protocol to identify encrypted data that contain a particular keyword. These methodologies ensure the privacy of the outsourced data and the search query, preventing the CSP from deducing any information about the outsourced data that can lead to a potential loss of privacy. An index-based data search employs a different methodology than an encrypted data search. Instead of executing a search query over encrypted data, the search query is evaluated for the index (inverted index) associated with the outsourced data. Trusted entities can be employed to persist the index and evaluating the search query. In contrast to that, index can be stored in cloud storage in encrypted form along with the outsourced data and concealed search queries can be used to search the cloud.

The aforementioned methodologies provide accessibility to relevant data and also ensure data privacy. However, these methodologies are fairly limited in their functionality and greatly affect the utility of cloud-based storage services. A search over encrypted data can only search for predefined keywords for which trapdoors are defined, and in the case of data sharing and collaborative services, these trapdoors are shared among subscribers. In an index-based data search, where the index is used by a trusted entity, the cloud storage is underutilized for only the outsourced data, whereas the search queries that are handled by a trusted entity can only retrieve the data that have an exact match between the search criterion and index entries. Similarly, when the index is outsourced to cloud storage, the CSP can learn the access patterns of subscribers and can deduce confidential information about the outsourced data and subscribers. For instance, if the outsourced data of a patient is searched by a medical doctor specializing in diabetes mellitus, this leads to a possibility that either the outsourced data contains information regarding diabetes mellitus or that the patient is suffering from diabetes mellitus.

Considering the limitations of conventional methodologies to efficiently retrieve outsourced data taking data privacy into consideration, there is a need for a searching methodology that can achieve semantic data retrieval and for an oblivious data search. Semantic data retrieval will ensure that relevant data can be discovered even if there is no exact match between the outsourced data and the search criteria defined by a subscriber. This will greatly increase the efficacy of the searching methodology in data sharing and collaboration services where the exact contents of the outsourced data are not known to participating subscribers, and only abstract ideas/concepts are communicated between them. For example, the employees of an insurance company who are collaborating on a task to define premium rates for next year’s insurance policy, do not know the actual contents of the survey reports shared by their colleagues. However, they want to determine if there are any surveys on viral diseases in a certain vicinity. An oblivious search will lead to maximized utilization of the cloud infrastructure without relying on a trusted third party and will enable the CSP to evaluate the encrypted search queries.

In this research, we propose an privacy-aware content discovery methodology that enables subscribers of a cloud storage service to locate relevant data contents without using actual keywords from the outsourced data. It is an index-based privacy-aware data searching methodology that does not rely on a trusted third party to evaluate the search query. It realizes privacy-aware content discovery, which ensures that only authorized subscribers are able to search the outsourced data. It also prevents the CSP and unauthorized subscribers from learning the presence or absence of any keywords and deducing information that can lead to a potential loss of privacy, encompassing the outsourced data and the subscribers’ personal information.

With the proposed methodology of privacy-aware content discovery, we make the following contributions in the area of cloud-based storage services:

- Privacy-aware search for encrypted data by utilizing semantic information to identify similarities between search criteria and outsourced data. The search criteria defined by a subscriber need not be exactly the same as in the outsourced data. If there exists a semantic relation between the search criteria and outsourced data, the relevant data contents can be retrieved;
- Privacy-aware data search without the need to share trapdoor information, and authorized subscribers can define their own search criteria. Their ability to access relevant data is not restricted to the information communicated by the data owner who outsources the data to the cloud storage;
- Maximized utilization of cloud storage services by persisting encrypted index and evaluated encrypted search queries within the domain of untrusted CSP; and
- Index and search query expansion by using semantic technologies to realize an encrypted data search similar to data contents for searching over the Internet.

The rest of this paper is organized as follows: Section 2 presents the related work. Section 3 defines the system design goals and the architectural and security model along with the assumptions. Section 4 is dedicated to the descriptive details of the proposed methodology. Section 5 discusses the implementation details, followed by evaluation of the results in Section 6. Section 7 presents the discussion on security, and Section 8 concludes the paper and discusses future directions.

## Related Work

In this section, we present methodologies to search encrypted data within an untrusted domain. Throughout this section, we mainly focus on cryptosystems, which exploit the mathematical properties of underlying cryptographic primitives to search encrypted data (i.e., trapdoor functions), and enterprise products, which define their protocols to match encrypted search queries and data. We mainly discuss the effects of these conventional methodologies on the efficacy and utility of cloud-based storage services within the context of data sharing and collaboration services.

Symmetric key cryptography (SKC) enables a search over encrypted data [10] by utilizing a trapdoor defined for a particular key only to identify a match between a search query (trapdoor function) and encrypted data. SKC has been used in various schemes for searching over encrypted data, in which trapdoors are used to identify a match between the index of encrypted keywords instead of encrypted data [13], [14], [15]. However, the basic principles of the trapdoor’s definition and the matching remain the same. A trapdoor-based search for public key cryptography (PKC) was proposed by Boneh et al. [11]. It leverages an untrusted server to search encrypted data using a public key, without the need to decipher concealed data. Schemes to search encrypted data that are based on SKC and PKC are limited in functionality because encrypted data can only be searched for keywords having corresponding trapdoors that are defined by the data owner who encrypts the data. Also, these trapdoors must be transmitted to authorized users, enabling them to access relevant data using search queries. Thus, methodologies relying on trapdoor-based cryptography assume guaranteed availability of the data owner or a trusted third party (TTP) to transmit a trapdoor to authorized users according to their access privileges.

To search confidential personal healthcare records, Li et al. proposed the Authorized Private Keyword Search (APKS) [12]. APKS utilizes Hierarchical Predicate Encryption (HPE) to realize a search over encrypted data [16] and employs a TTP to distribute capabilities (trapdoors) to authorized users according to their access privileges. These capabilities are then submitted to the CSP to evaluate the search query. Wang et al. proposed a methodology to rank search results according to their relevance with the selection criteria (trapdoor) [17]. However, it only supports a single trapdoor-based search query, greatly reducing its efficacy in defining the complex selection criterion and lacking the realism to search a large amount of data. A Searchable Cryptographic Cloud Storage System (CS2) focusing on dynamic data updates also provides a search over encrypted data [18]. Instead of searching the entire encrypted data repository, CS2 utilized the inverted index. However, CS2 is limited to cloud-based storage services and is not applied for cloud-based data sharing and collaboration services. Recently, Wenhai Sun et al. presented a privacy-preserving multi-keyword text search (MTS) with similarity-based ranking [19]. MTS utilizes tree-based indexing with adaption methods for a multi-dimensional algorithm. It ensures the confidentiality of the search query and the index data structure. However, it assumes that the user searching the cloud storage always behaves honestly, whereas the cloud server is honest but curious. This assumption can greatly affect the practicality of MTS for cloud-based storage services, focusing on data sharing and collaboration, in which users can behave maliciously to determine the presence or absence of a particular keyword(s). Oblivious Term Matching (OTM) realizes an encrypted index search, where the index is computed over encrypted outsourced data. OTM obliviously evaluates encrypted search queries, where it does not consider relevant data access with consideration of semantic enrichment of the encrypted index or search queries [20]. The proposed methodology of semantic data search uses OTM to identify similarities between search criteria and outsourced data.

To achieve efficient data retrieval over large data contents, enterprises rely on search products that are customized to their specific needs and requirements. The Google search appliance [21] and Windows enterprise search products [22] offer such search solutions. These products create a searchable centralized enterprise-wide index that is used within the enterprise’s data center or can be configured to use cloud repositories. The search queries are evaluated and the results are filtered according to the access privileges of a user. Since these products evaluate access privileges after the execution of a search query, they require search services to be hosted within the federated domain of an enterprise. Thus, these search services retrain the migration of an enterprise-to-cloud ecosystem as it has to engage its own dedicated computation and storage resources for customizable search services. The authors in [23] have shown that, by carefully modeling search queries, malicious users can deduce confidential information from the centralized index, even if their access privileges do not allow them to access encrypted data.

The aforementioned methodologies for searching encrypted data focus on the confidentiality of the search query and the outsourced data. However, these methodologies do not consider the privacy of the query evaluation process that is employed to identify the relevant data contents. It can be exploited by a malicious CSP to deduce information that can lead to a potential loss of privacy. In cloud-based data sharing services, if multiple users are searching for a similar keyword, the CSP can effortlessly identify the importance of the outsourced data and can concentrate its malicious intents to deduce confidential information from the data. For instance, if the employees from the accounts and planning departments of an organization are searching data that has been outsourced to a folder called projected income statements, the CSP can determine the irregularity in access patterns and consequently affect the highly sensitive stock market, thereby disrupting the stock prices. Thus, a methodology that can obliviously search cloud-based repositories is of great importance, as it restrains the capability of a CSP to deduce or infer confidential information.

**Fig 1:**
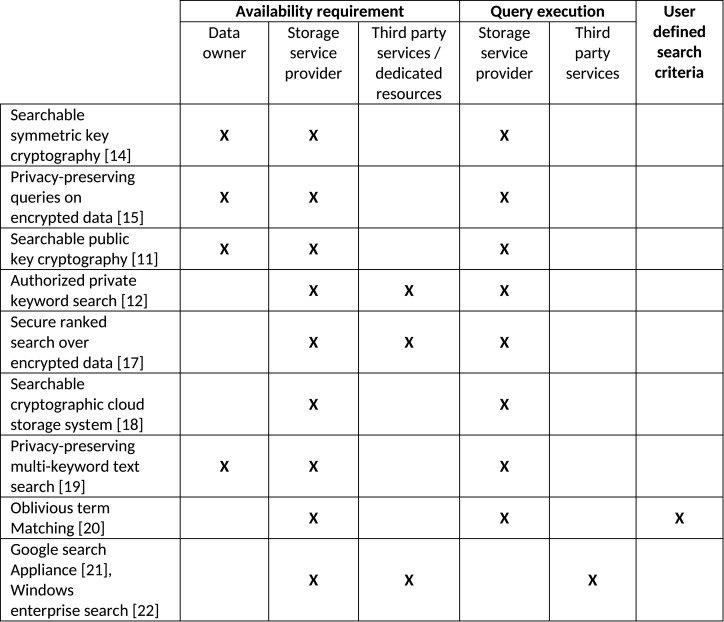
Features of conventional encrypted data search methodologies.

Fig 1 highlights the important features of existing methodologies for encrypted data search i.e., availability requirement for involved entities, entity responsible for evaluating the search query, and capability of a user to define arbitrary search queries. Although these methodologies realize encrypted data search, however their functionality is limited to exact matching between the search query and encrypted data i.e., trapdoors and encrypted index. Also these methodologies restrain authorized users to define their own search queries. In the subsequent sections, relevant data access with semantically enriched search queries is presented. The proposed methodology realizes semantic search enabling authorized users to define their own conjunctive search queries without compromising privacy of the outsourced data and search queries as well.

## Design Goals, System and Security Model, Main Idea, Assumptions and Notations

### System Design Goals

A data search within a cloud storage service allows subscribers to locate the required data contents. However, when data is outsourced to an untrusted domain of a public cloud service provider in encrypted form, standard search queries do not work, as the search criteria cannot be mapped to encrypted data. These search queries can also reveal confidential information about the outsourced data and the data owner. The design goal of our proposed system is to allow the subscribers of a public cloud storage service to search encrypted data in a similar way as contents are discovered over the Internet. However, search queries should not reveal any information to the cloud service provider, which can lead to the potential loss of privacy, affecting the outsourced data and personal information.

### System Model

To search encrypted data similar to content discovery works over the Internet, the public cloud storage service provider, repository owner, and content contributor are considered as the involved entities. For the sake of simplicity in the subsequent descriptive details, we refer to these entities as the cloud server, owner, and subscriber, respectively. The cloud server owns the cloud infrastructure (i.e., storage, computation, and network) and provisions its access on a subscription basis. The owner is a cloud storage subscriber who creates a shared repository that is accessible to other authorized subscribers. Subscribers contribute to the shared repository by outsourcing data contents. The owner and authorized subscribers search the cloud storage (shared repository) by submitting search queries to the cloud server. Search queries are obliviously evaluated by the cloud server, and the search results are provided to the respective entity according to its access privileges.

### Security Model

We consider the cloud server to be an untrusted entity that can collude with unauthorized subscribers to compromise the privacy of the outsourced data. It can assist unauthorized subscribers to search the outsourced data. Since the search query is evaluated by the cloud server, its result can be exploited to deduce confidential information about the outsourced data. To ensure the confidentiality of the outsourced data, only encrypted data is outsourced to the cloud server. In addition, to prevent the cloud server from inferring confidential information about the outsourced data, the encrypted search query is obliviously evaluated. This restrains the cloud server and unauthorized subscribers from learning of the presence or absence of a particular word or concept in the outsourced data.

### Main Idea

Suppose the Daily News is a nationwide newspaper that provides coverage of national and international events. Alice is a subeditor working for the Daily News. She oversees the department that focuses on financial corruption. At any particular point in time, she is working on multiple cases. She has assigned evidence collection and report compilation tasks to her subordinate journalists. To deal with the problem of content accessibility on her office and mobile devices, she has subscribed to a public cloud storage service that is provisioned by Eve. Since her subordinate journalists share confidential information with her, she does not want Eve to learn or deduce any information about the outsourced data. To ensure the privacy of the data, each journalist outsources encrypted data to the repository shared by Alice.

Bob and Mallory are Daily News journalists who work with Alice. Bob is an expert at retrieving information from online resources. Mallory’s expertise is in finding the ground truth by contacting the concerned authorities. Both are directed to submit their findings on a financial scam that was recently exposed by the Fraud and Financial Crime Division of the State. Alice has provisioned access to both Bob and Mallory to a cloud-based shared repository. Bob retrieves all of the related information from online resources, whereas Mallory compiles her report using the information she has collected from the appropriate authorities. Before outsourcing their findings to a shared repository, they index the information. The index is then further enhanced by augmenting it with missing relevant information. After that, the findings and the enhanced index are encrypted and outsourced to the shared repository.

Whenever Alice needs to search for a file containing particular information, she defines a search criterion. The search criterion is then enriched by adding missing relevant information. The augmented search criterion is then encrypted with the secret key, and after that, Alice models an oblivious search query using the encrypted search criterion. The oblivious query is then submitted to the cloud server, which replies with the response.

**Fig 2:**
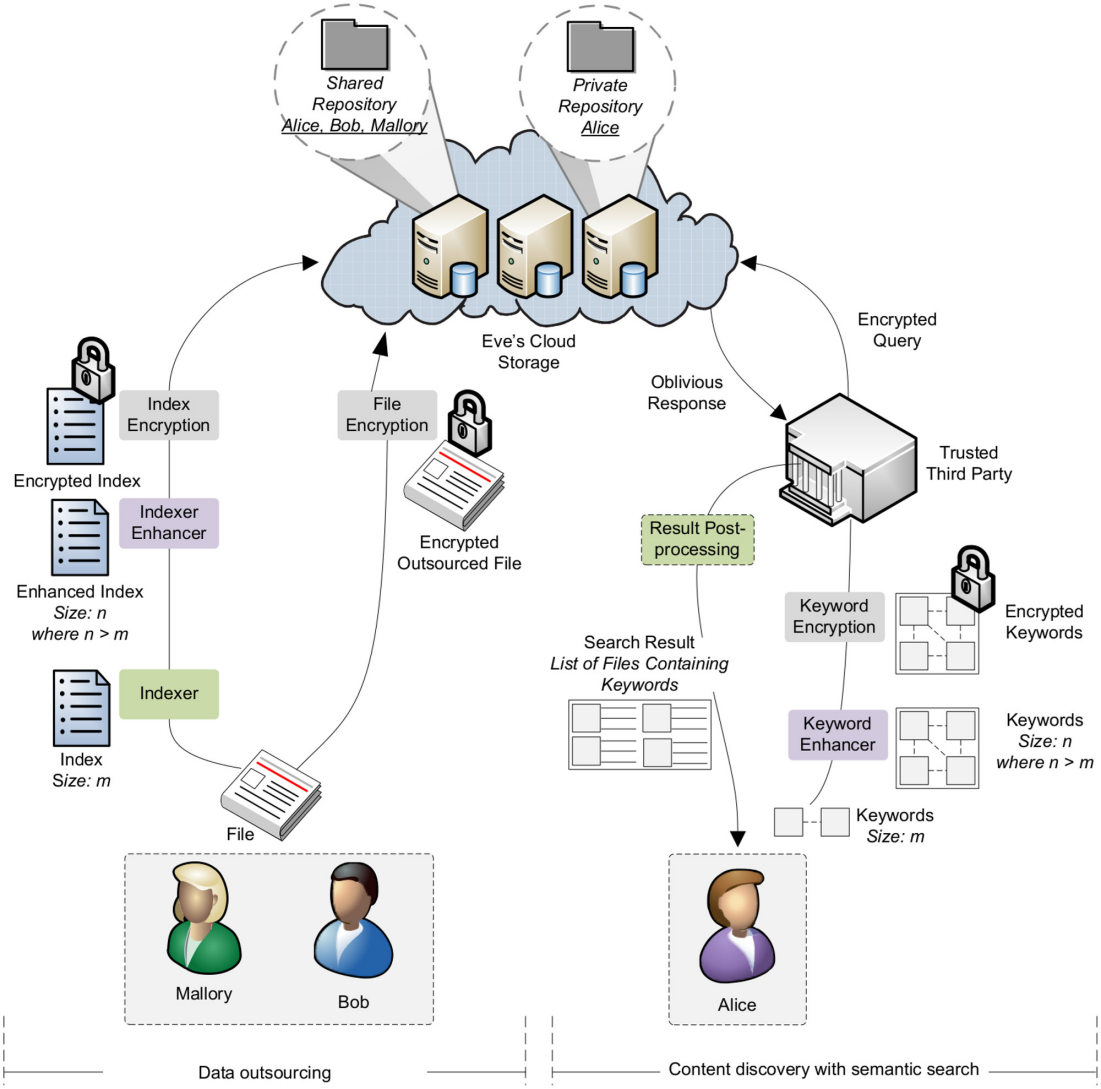
Semantic search over encrypted data—conceptual model.

Alice processes the cloud server response and determines the presence / absence of keywords that were defined in the search criterion. From the processes of query formation, evaluation, and post-processing of the result, the cloud server learns nothing about the outsourced data or the search query; the search evaluation is oblivious to the cloud server. If an unauthorized subscriber tries to search the repository, the proposed system generates a randomized response. Fig 2 illustrates the conceptual model of our proposed system for searching encrypted data in an untrusted domain.

### Assumptions and Notations

The proposed system focuses on semantic search for encrypted data. We assume that the owner has shared a symmetric encryption key with the authorized subscribers. The data that is outsourced to a shared repository is always encrypted with that key. In the subsequent descriptive details of the proposed system, the specifics of sharing data within an untrusted domain are intentionally neglected for the sake of simplicity. Readers can refer to [24] and [25] for descriptions of efficient and secure data sharing within public cloud storage services. The proposed system address the problem of privacy-aware relevant data access in untrusted cloud storage services. Ensuring data integrity and correctness is beyond the scope of research undertaken in this work; interested reader can refer to [26] for more details on public auditing.

Table 1 presents the notations used in the descriptive details of our proposed system to semantically search the encrypted data. F represents a file that is outsourced to cloud storage. I stands for an index computed over F that contains the keywords and their respective frequencies i.e., $I=\{\langle kw_{0},f_{0}\rangle \ldots \langle kw_{n},f_{n}\rangle \}$, where *n* is the size of the index. $I_{s}$ represents a semantic index that is generated by identifying synonyms and the root word for each $kw_{0\ldots n}:kw_{i}\in I$ i.e., $I_{s}=\{\langle kw_{0},syn_{0_{0\ldots \nu }},rw_{0},f_{0}\rangle \ldots \langle kw_{n},syn_{n_{0\ldots \nu }},rw_{n},f_{n}\rangle \}$, where *syn*_*i*_0…*ν*__ is the list of synonyms of *kw*_*i*_, and *rw*_*i*_ is its root word. H is an encoding function that is publicly known and encodes variable sized keywords into integer values of fixed length. $E_{H}$ and $D_{H}$ are homomorphic encryption algorithms. *σ*_*pk*_ and *σ*_*sk*_ are public and secret keys respectively, that are used by homomorphic encryption algorithms. These algorithms enable the processing of encrypted values (search query and encrypted index) without the need to decrypt them. $E_{S}$ and $D_{S}$ are symmetric encryption algorithms with a secret key *k*. F and $I_{s}$ are encrypted with symmetric encryption algorithms before they can be outsourced to a cloud server. $E_{A}$ and $D_{A}$ are asymmetric encryption algorithms associated with *k*_*pub*_ and *k*_*pri*_ public and private keys, respectively. *α*_0…*n*_ represents a list of polynomial coefficients that are used to formulate an oblivious search query. Δ_*y*_0…*n*__ is a list of oblivious values that are obtained as a result of the oblivious search query execution by the cloud server.

**Table 1:** Notations used in the descriptive detail of semantically enriched encrypted data search.

*Notation*	*Description*
F	File outsourced to a shared repository.
$I=\{\langle kw_{0},f_{0}\rangle \ldots \langle kw_{n},f_{n}\rangle \}$	Index file that contains *n* keywords.
$I_{s}=$ {〈*kw*_0_, *syn*_0_0…*ν*__, *rw*_0_, *f*_0_〉…〈*kw*_*n*_, *syn*_*n*_0…*ν*__, *rw*_*n*_, *f*_*n*_〉}	Semantic index—an enriched form of I. *kw* is a keyword from I, *syn*_0…*ν*_ is a list of its synonyms and *rw* is its root/parent word.
H	Publicly known encoding function that transforms an arbitrary-sized string to an integer value of *q* modulo, where *q* is a large prime.
$E_{H}$, $D_{H}$	Homomorphic encryption and decryption algorithms.
*σ*_*pk*_, *σ*_*sk*_	Public and secret key pair for homomorphic encryption algorithms.
$E_{A}$, $D_{A}$	Asymmetric encryption and decryption algorithms.
*k*_*pub*_, *k*_*pri*_	Public and private key pair for asymmetric encryption algorithms.
$E_{S}$, $D_{S}$	Symmetric encryption and decryption algorithms.
*k*	Secret key of symmetric encryption algorithms. It is shared with authorized users only.
*α*_0…*n*_	List of coefficients of a polynomial *P* which defines a search query.
Δ_*y*_0…*n*__	List of oblivious values generated as a result of query execution by the cloud server.

## Proposed System

The proposed methodology of encrypted data search based on semantically enriched index and search queries is presented in this section. It is divided into five cohesive steps: indexing, data outsourcing, query formulation, query execution, and post-processing of results.

### Indexing

A semantic search over encrypted data is achieved by evaluating search queries for an enriched inverted index ($I_{s}$) associated with the outsourced data (F). Since, we want subscribers to search outsourced data using search queries that are semantically equivalent, the inverted index (I) is augmented with extra information. This extra information enables us to identify outsourced data that contains relevant information instead of finding an exact match between the search query and the keywords extracted from the outsourced data.

To achieve this, the indexing is further divided into two phases. In the first phase, for each F that needs to be stored in cloud storage, I is generated. It contains all of the keywords (*kw*_0_…*kw*_*n*_) that appear in F, along with their respective frequencies i.e, $I=\{\langle kw_{0},f_{0}\rangle ,\ldots \langle kw_{n},f_{n}\rangle \}$. After that I is further processed to augment it with semantic information. For that, $kw_{0\ldots n}:kw_{i}\in I$ are searched in a lexical database. This enables us to identify synonyms (*syn*_0…*ν*_) of *kw*_*i*_ that do not exist in I but where *syn*_*i*_0…*ν*__ and *kw*_*i*_ semantically equivalent. Further root word (*rw*_*i*_) of each *kw*_*i*_ is also extracted from the lexical database. *rw* assists us in finding the keywords that share the same root word, consequently identifying the relevancy between the search query and the encrypted outsourced data. Once *syn*_0…*ν*_ and *rw* are identified, I is augmented with this extra information and is transformed into semantic index i.e., $⊎\left(I,syn_{0\ldots \nu },rw\right)\to I_{s}$; where ⊎(⋅) is a function that appends *syn*_0…*ν*_ and *rw* to I removing any duplicate values, where $I_{s}=\{\langle kw_{0},syn_{0_{0\ldots \nu }},rw_{0},f_{0}\rangle \ldots \langle kw_{n},syn_{n_{0\ldots \nu }},rw_{n},f_{n}\rangle \}$.

### Data Outsourcing

To ensure that the cloud server cannot exploit $I_{s}$ by deducing confidential information about the outsourced data. $I_{s}$ is concealed using a symmetric encryption algorithm before it can be outsourced to a cloud server. The secret key (*k*) for the symmetric encryption algorithm is shared among all of the authorized subscribers by the owner who having ownership rights over the shared cloud based repository. The scope of this paper is limited to encrypted data search, readers may refer to [27] for more details secret key sharing and user revocation in untrusted domain.

**Fig 3:**
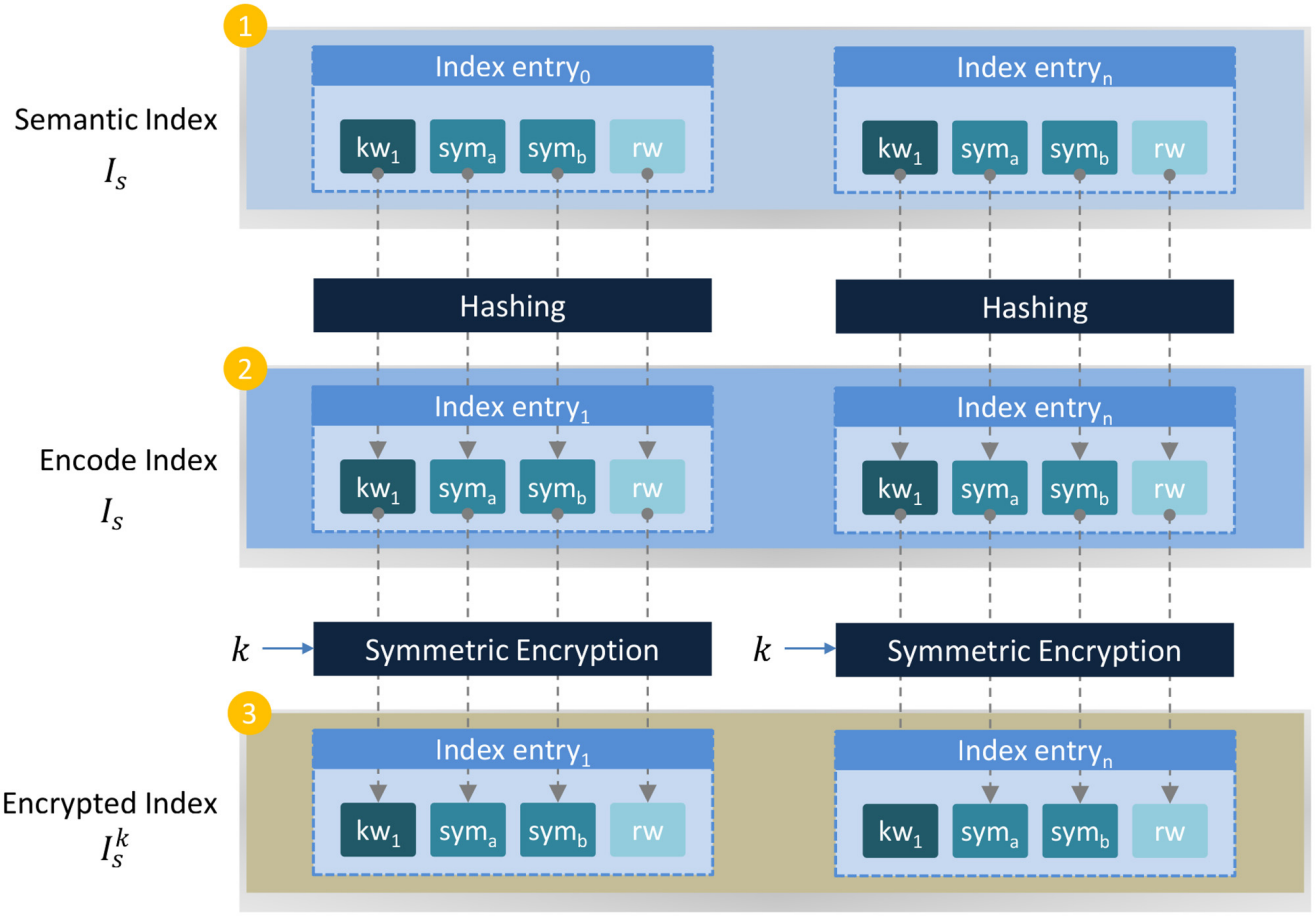
Encoding semantically enriched index and securing its confidentiality through symmetric encryption.

In order to ensure that the search query can be obliviously evaluated by the cloud server, the owner encodes each keyword ($kw_{i}\in I_{s}$) using a publiclly known encoding function $H\left(I_{s}\right)\to {\hat{I}}_{s}$. ${\hat{I}}_{s}$ is then encrypted using a symmetric encryption algorithm $E_{S}({\hat{I}}_{s},k)\to {\hat{I}}_{s}^{k}$. Once the confidentiality of $I_{s}$ is ensured, ${\hat{I}}_{s}^{k}$ along with $F^{k}$ are outsourced to the cloud server. Since, *k* is only shared among the authorized subscribers and the owner, unauthorized subscribers cannot deduce any information about the outsourced data, even if they conspire with the cloud server. Fig 3 illustrates the entire process of securing inverted index with symmetric encryption.

### Query Formulation

To search encrypted data, a subscriber defines a search criterion ($C_{kw_{0\ldots j}}$), which consists of a set of keywords (*kw*_0…*j*_) that are used to search the relevant encrypted outsourced data. Since, we want to realize a semantic search over encrypted data, the search criteria defined by the subscriber is further enriched by identifying synonyms of $kw_{0\ldots j}:kw_{i}\in C_{kw_{0\ldots j}}$. Once the relevant keywords (*syn*_0…*ν*_) are identified $C_{kw_{0\ldots j}}$ is enriched by adding *syn*_0…*ν*_ to $C_{kw_{0\ldots j}}$ i.e., $⊎\left(C_{kw_{0\ldots j}},syn_{0\ldots \nu }\right)\to C_{kw_{0\ldots l}}$, where *j* < *l*, and ⊎(⋅) is a function that appends *syn*_0…*ν*_ to $C_{kw_{0\ldots j}}$.

Since, search queries are evaluated by the cloud server, there is a need to conceal $C_{kw_{0\ldots l}}$ using an appropriate symmetric encryption algorithm. To prevent the cloud server from deducing any information about the encrypted outsourced data, the owner encodes $C_{kw_{0\ldots l}}$ using a publicly known encoding function. For example, $H\left(C_{kw_{0\ldots l}}\right)\to {\hat{C}}_{kw_{0\ldots j}}$—H(·) must be the same encoding function as that used in the data outsourcing; otherwise, an oblivious search query cannot be successfully evaluated. After that ${\hat{C}}_{kw_{0\ldots l}}$ is encrypted with the symmetric encryption i.e., $E_{S}\left({\hat{C}}_{kw_{0\ldots l}},k\right)\to {\hat{C}}_{kw_{0\ldots l}}^{k}$, where *k* is a shared symmetric encryption key, which is the same as that used in the data outsourcing to conceal ${\hat{I}}_{kw_{0\ldots n}}$.

To this stage, ${\hat{C}}_{kw_{0\ldots l}}$ has been concealed, however in order to realize an oblivious query evaluation there is a need to further process ${\hat{C}}_{kw_{0\ldots l}}^{k}$. A polynomial (*P*(*x*)) is defined such that the concealed $kw_{0\ldots n}:kw_{i}\in {\hat{C}}_{kw_{0\ldots l}}^{k}$ are the root of *P*(*x*) i.e., $P\left(x\in {\hat{C}}_{kw_{0\ldots l}}^{k}\right)=\sum_{i=0}^{l}\alpha_{i}x^{i}=0$, where *α*_0…*l*_ are the coefficients of *P*(*x*).

**Fig 4:**
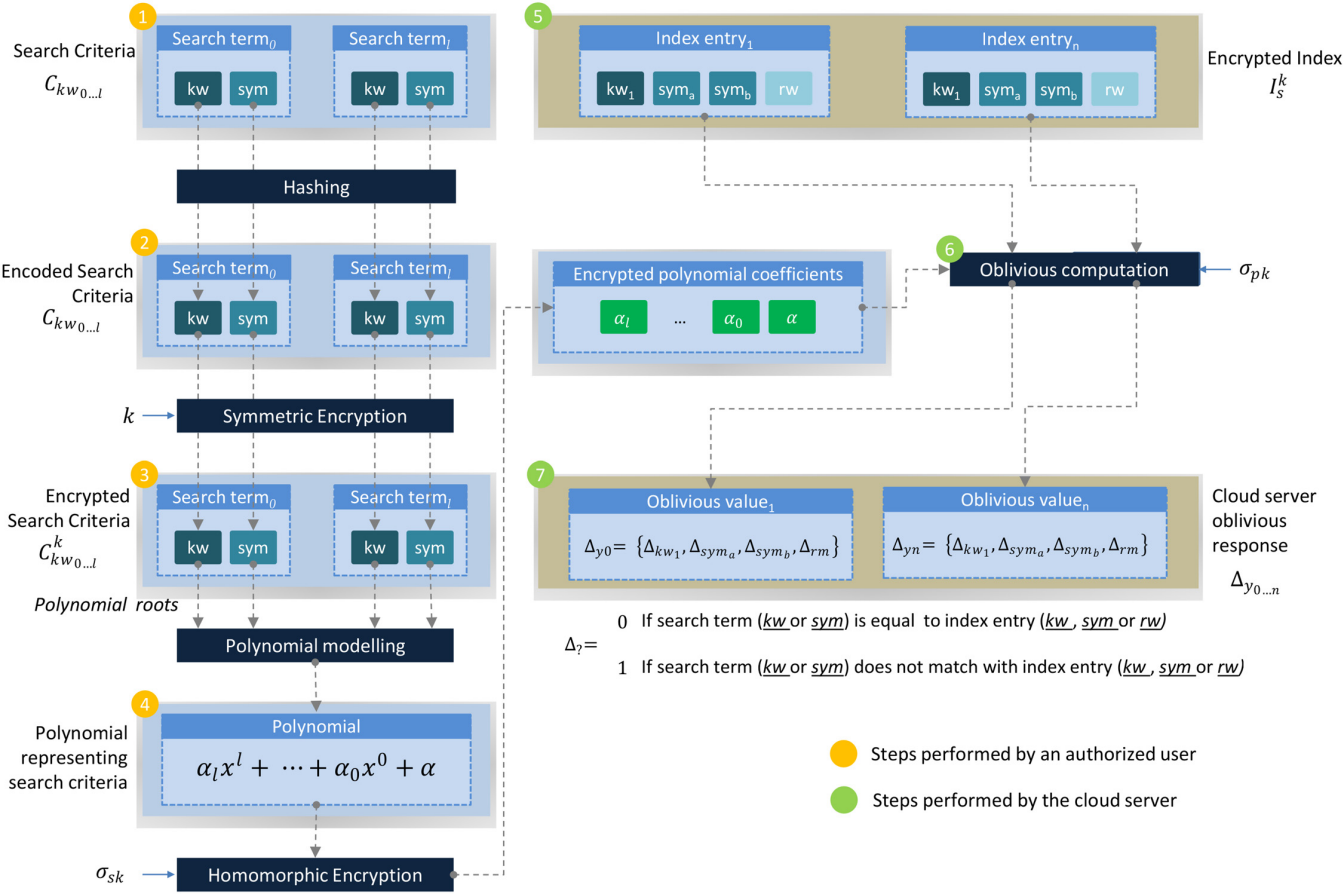
Encoding semantically enriched search criteria and modeling search query for oblivious computation.

Once the polynomial *P*(*x*) has been defined, a homomorphic encryption key pair (*σ*_*pk*_, *σ*_*sk*_) is initialized. Homomorphic encryption enables the cloud server to process the encrypted search query and also restrains its ability to learn the result of the query evaluation. After that, *α*_0…*l*_ are encrypted i.e., $E_{H}\left(\alpha_{0\ldots l},\sigma_{sk}\right)\to \alpha_{0\ldots l}^{\sigma_{sk}}$, $\alpha_{0\ldots l}^{\sigma_{sk}}$ along with *σ*_*pk*_ are transfered to the cloud server. $\alpha_{0\ldots l}^{\sigma_{sk}}$ are used as the encrypted search query whereas *σ*_*pk*_ enables the evaluation of an encrypted search query without the need to decipher ${\hat{I}}_{s}^{k}$ and $\alpha_{0\ldots l}^{\sigma_{sk}}$. Fig 4 describes the entire process of query formation.

### Query Execution

To semantically identify the encrypted data, the search query is obliviously executed by the cloud server. $\alpha_{0\ldots l}^{\sigma_{sk}}$, which is submitted by a subscriber, is evaluated for ${\hat{I}}_{s}^{k}$. By using *σ*_*pk*_, for $kw_{0\ldots n}:kw_{i}\in {\hat{I}}_{s}^{k}$, the cloud sever computes the oblivious value i.e., $\Delta_{y_{0\ldots n}}=r.P\left(y_{i}\in {\hat{I}}_{s}^{k}\right)$, where $y_{i}=kw_{i}\in {\hat{I}}_{s}^{k}$ and *r* is a random number. The computation of the oblivious value ensures that the owner can identify the match between $\alpha_{0\ldots l}^{\sigma_{sk}}$ and ${\hat{I}}_{s}^{k}$. Fig 4 describes the oblivious query execution process.

Since, we are employing homomorphic encryption, the cloud server cannot learn whether $kw_{i}\in {\hat{I}}_{s}^{k}$ is a root of $\alpha_{0\ldots l}^{\sigma_{sk}}$. Thus, it cannot identify a match between the encrypted search criterion and the encrypted index. Once Δ_*y*0…*n*_ = *r*.*P*(*y*_0…*n*_) are computed, the cloud server transfers the result of the search query evaluation to the subscriber.

### Post-processing of results

The oblivious values that the subscriber receives from the cloud server can only be deciphered using the valid homormophic key, which is the secret key, *σ*_*sk*_. This ensures that the cloud server cannot collude with malicious subscribers to exploit the oblivious query evaluation process. On receiving the cloud server’s response, the subscriber deciphers Δ_*y*_0…*n*__ i.e., $D_{H}\left(\Delta_{0\ldots n},\sigma_{sk}\right)=\psi_{0\ldots n}$, where *ψ*_*i*_ can be a zero or non-zero randomized value.

Since, the search query $\alpha_{0\ldots l}^{\sigma_{sk}}$ submitted by the subscriber is constituted of root values from ${\hat{C}}_{kw_{0\ldots l}}^{k}$ the decryption of *ψ*_*i*_ turns out to be zero for all those $y_{i}=kw_{i}\in {\hat{I}}_{s}^{k}$ that are equal to the root value of *P*(*y*_*i*_), i.e., $kw_{i}\in {\hat{C}}_{kw_{0\ldots l}}^{k}\wedge kw_{j}\in {\hat{I}}_{kw_{0\ldots n}}^{k}$ where *kw*_*j*_ = *kw*_*i*_. For all other values where *kw*_*j*_ ≠ *kw*_*i*_, *ψ*_*i*_ would turn out to be a random value (see Eq 1).

$$P\left(y\right)=\overset{l}{\underset{i=0}{\sum }}\alpha_{i}y^{i}\{\begin{array}{ll} =0 & \text{if }y\text{ is root of }P\left(y\right).\\ \neq 0 & \text{a random value }r\text{ for all other index entries.} \end{array}$$ (1)

Thus, only by deciphering Δ_*y*0…*n*_ with valid *σ*_*sk*_ owner can learn the result of encrypted search query. However, for the cloud server the evaluation of the search query will remain oblivious.

## Implementation

**Fig 5:**
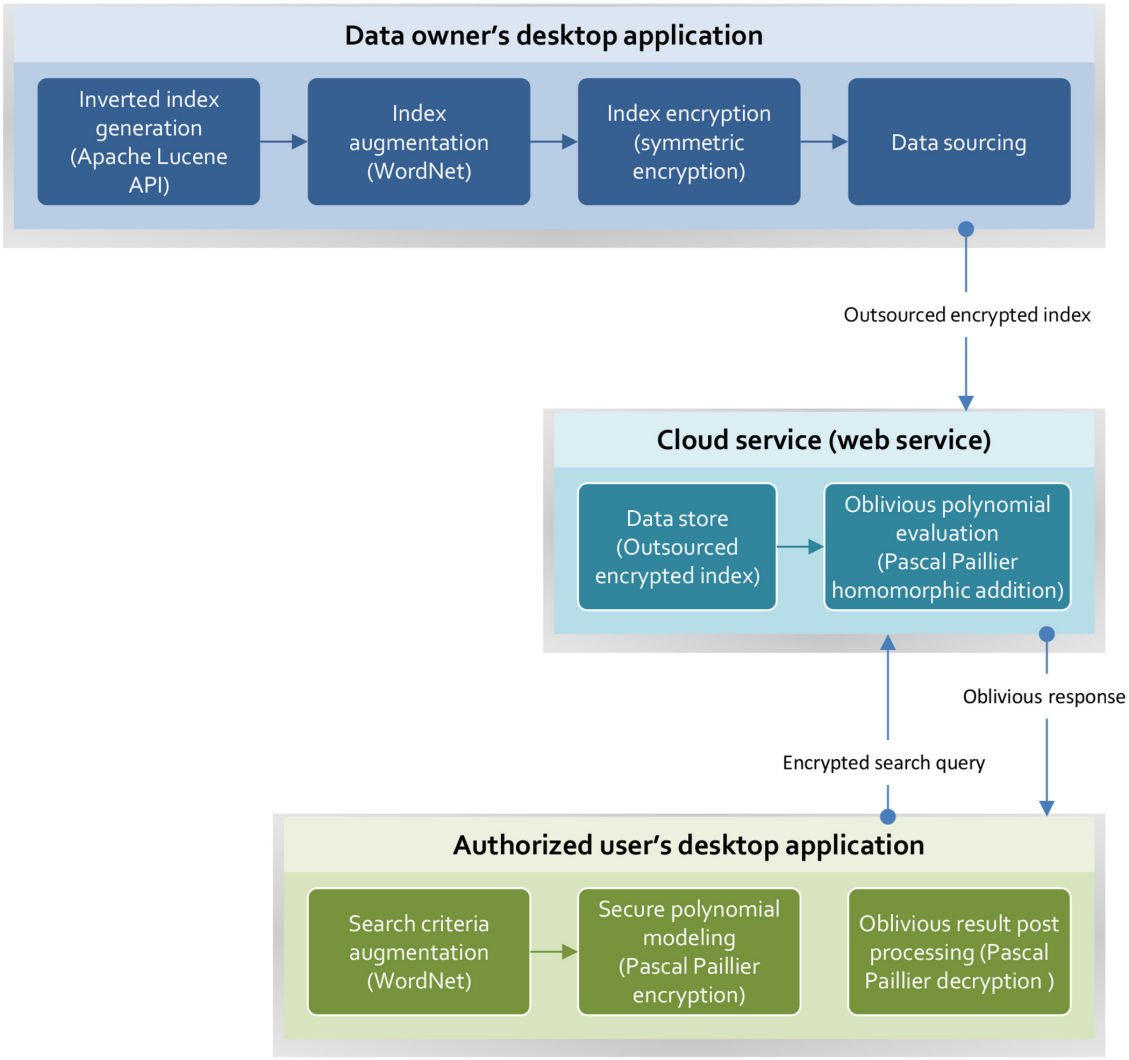
Core functionalities—desktop application and web service.

The proposed methodology for a semantically enabled search of encrypted data is realized using jdk 1.7. We implemented a Java based desktop application and web service. The desktop application performs keyword extraction, indexing and search query augmentation, and post-processing of the result, whereas the web service is solely responsible for the oblivious evaluation of the encrypted search queries. Fig 5 illustrates the core functionalities of desktop application (data owner and authorized users) and web service.

In desktop application for data owner we generates an inverted index from the plain text, i.e., the data that needs to be outsourced to cloud storage. For this, we employ Apache Lucene API [28], which is a fully-featured text search engine that is focused on high performance. Apache Lucene enables us to extract all keywords, avoiding indexing of the stop word and repeated keywords. Once the keywords are extracted from the plain text in the form of the inverted index, we augment them with semantic information, i.e., synonyms and root words, using WordNet [29]. To use the augmented keywords to search the encrypted data, a hash of the individual keywords is computed using the SHA-512 hashing algorithm. The hashed keywords are then encoded into BigInteger values of arbitrary size. Once encoded, the inverted index entries are encrypted with the symmetric encryption algorithm and are outsourced to the web service.

User desktop application authorized users model their search query in the form of a polynomial and learn the semantic map between their search criteria and the outsourced encrypted index. The search criteria defined by a user is augmented and encoded in a similar way, as discussed for the inverted index. To evaluate the encrypted search query, we utilize the Pascal Paillier cryptosystem [30]. The secret key of Pascal Paillier is used to conceal the search criteria, whereas a public key is used by the cloud server to evaluate the search query. For each encrypted keyword in the encrypted index, the search is evaluated and the result is transmitted back to the user.

## Evaluation

The proposed methodology for the encrypted data search was evaluated on a 2.60 GHz Windows 7 PC with 2.0 GB of main memory. We opted for a relatively low-end machine to demonstrate that the proposed methodology can be realized for any public cloud-based storage service, since it does not have any special computational requirements.

In the subsequent section, we first present the computational complexity of the semantic search for encrypted data. We then discuss the computational analysis of augmenting the inverted index and the search criteria with the semantic information. In the last section, we show the computational load of the oblivious query evaluation, where the search query is composed of multiple search criteria.

### Complexity Analysis

The computational complexity and the amount of data transmitted between the entities are analyzed in order to illustrate the efficacy of our proposed encrypted data search. Both of these parameters are directly proportional to the size of the encrypted index outsourced to the cloud storage and the size of the encrypted search query. Table 2 shows the set of operations performed in each step of our proposed methodology, along with the input size and the amount of transmitted data. For the sake of simplicity, we regarded the cryptographic and hashing operations as constant time operations, because the proposed methodology is not confined to any particular encryption or hashing algorithm.

**Table 2:** Complexity analysis of semantic search for encrypted data.

Steps	Operations	Input Size	Computational Complexity	Transmitted Values
Indexing	Public encoding & Symmetric encryption	*N*	*O*(*N*)	–
Data outsourcing	–	*N*	*O*(*N*)	*N*
Query formulation	Asymmetric encryption & Polynomial modeling	*n*	*O*(*n*^3^)	*n* + 2
Query execution	Polynomial evaluation	(*n* + 1)*N*	*O*(*n*^2^.*N*)	*N*
Post-processing of results	Asymmetric decryption	*N*	depends on *n*	depends on *n*

**Indexing:** To extract keywords and to identify the semantically equivalent words for each extracted keyword, we utilize freely available libraries, such as Apache Lucene and Wordnet. Since these libraries complement our proposed system, we consider their execution at a constant time. Thus, the computational complexity of the index is *O*(*N*), where *N* is the size of the augmented index containing both the synonym and the root word.

**Data outsourcing:** We regard the computational complexity of the data outsourcing to be *O*(*N*), where *N* is the size of the augmented index. In total, *N* values are transmitted to the cloud server.

**Query formulation:** The query formation is comprised of two steps. In the first step, the user defines the search criteria, which is then expanded with semantic information and finally encoded into a fixed length integer value using a hashing algorithm. In the second step, encoded values are used to model a polynomial, which is then concealed using the Pascal Paillier homomorphic encryption algorithm. Since the retrieval of the synonyms and root word and the encoding of the expanded search criteria are regarded as constant time operations, the computational complexity of the first step is *O*(*n*), where *n* is the size of the expanded search criteria. For the second step, the first individual encoded search criterion is modeled as a polynomial (where the search criterion is a root of the polynomial), the individual polynomials are multiplied together, and the coefficients of the resultant polynomial are concealed with the private key of the homomorphic encryption algorithm. Since the encrypted search query is modeled in three steps, its computational complexity is *O*(*n*^3^), where *n* is the size of the encoded search criteria. In total, *n* + 2 values are transmitted to the cloud server, where *n* + 1 is the number of coefficients, and there is one public key of the homomorphic encryption algorithm.

**Query execution:** The encrypted search query is evaluated for each encrypted entry in the index outsourced to a cloud server. The computation complexity of the query execution depends on two factors: the size of the index, *N*, and the size of the polynomial that models the search query, *n* + 1. Thus, the computational complexity of an oblivious search query evaluation in terms of the Big-*O* notation can be expressed as *O*(*n*^2^ *N*). The size of search query results is also directly proportional to the size of the index. In total, *N* values are transmitted to the user as a result of the search query execution.

**Post-processing of results:** Post-processing of the results is relatively a simple process, and it only deciphers the number of oblivious values, *N*, sent by the cloud server. Since we consider the computational load of the cryptographic operations as a constant, the computational complexity of the post-processing of the result can be regarded as *O*(*N*). The computational time required to post-process an individual oblivious record depends on size of search query i.e., number of keywords used to model conjunctive search query.

### Computational Analysis

The computational time required to enrich the inverted index and the search query with semantic information is presented along with the amount of time required to model an encrypted search query. We studied the computational time of conjunctive search queries and presented the time required to evaluate those encrypted search queries over the enriched inverted index. Table 3 shows the average computational time of the aforementioned steps computed over 100 iterations. To measure computational time we used Java time logging mechanism. System time to the precision of nanoseconds was logged at the beginning and end of the process, difference between logged timestamps was regarded as the time required to completely execute the process.

**Table 3:** Computational time analysis of semantic search for encrypted data.

Query Size (No. of keywords)	Query formulation (ms)	Query execution (ms)
2	238	245
4	411	791
6	590	1169
8	778	2811
10	982	4230
12	1187	6018
14	1405	8796

**Synonym identification:** The index and search query expansion are two important steps which enable a semantic search over encrypted data. For the computational analysis, we evaluated Wordnet API over a batch of 50 words. These words can be regarded as keyword entries in the inverted index and search criteria defined by authorized subscribers. For a batch, the total number of synonyms and the execution time are noted first, and we extracted 872 synonyms in 408 ms. This total number of synonyms is then divided by 50 to calculate the average number of synonyms per word, which is approximately 18 synonyms per word. Finally, the time required to extract these average synonyms per word is calculated as the total execution time divided by the total time multiplied by the average number of synonyms per word, i.e., (408/872) * 18 = 8.42 ms, which represents the average execution time per word. This exercise is repeated over 10 batches of different word sets for a more realistic time calculation. For the evaluation, we selected the standard implementation of WordNet and did not consider an optimization strategy.

**Query formulation:** The query formulation is comprised of the polynomial modeling and the asymmetric encryption of polynomial coefficients. As discussed in the complexity analysis, the computational cost of the query formulation depends upon the size of the search criteria that constitutes the encrypted search query. Unlike the conventional methodology, the proposed method supports a conjunctive search query, allowing authorized subscribers to set multiple search filters instead of relying on a single search criterion. Table 3 shows the average computational cost to model an encrypted search query with multiple search criteria.

**Query execution:** For each index entry (encrypted keyword), the cloud server evaluates the search query. The query evaluation is merely a process of polynomial evaluation at a certain value, and that value happens to be an individual keyword in the outsourced enhanced index. The computational time of the query execution depends on the size of the enhanced index and the encrypted search query. The entire process of query execution utilizes the homomorphic property of the Pascal Paillier cryptosystem. The result of the query execution is oblivious to the cloud server. The computational time of the encrypted search query comprises a range of two to ten search criteria, as shown in Table 3, which shows how the increase in the number of search criteria affects the computational time required to obliviously execute a search query.

## Security Analysis

In this section, we present the security analysis of our proposed methodology. Particularly, we focus on the capabilities of malicious entities to learn the encrypted search query and to deduce confidential information about the encrypted outsourced data. We examine the advantage of an untrusted cloud service provider to learn the result of the search query evaluation and to deduce information that can lead to a potential loss of privacy. We then discuss the scenario in which an unauthorized subscriber attempts to search encrypted data to which it does not have access.

The proposed methodology utilizes a number of cryptographic primitives to ensure execution of the encrypted search queries and to restrain malicious entities from deducing information that assists them in compromising the privacy of the outsourced data. As illustrated in the descriptive details of our proposed methodology, the inverted index is encrypted with symmetric encryption. To ensure oblivious evaluation of the search queries, homomorphic encryption is utilized along with a private matching protocol [31]. For the security analysis of these cryptographic primitives, readers can refer to [30] and [32]. In the subsequent sections, we examine the capabilities of malicious entities to deduce confidential information within the context of a semantic search over encrypted data.

### Malicious Cloud Server

The proposed methodology for encrypted data search utilizes the computational power and storage facility of a cloud server to execute search queries, instead of relying on a trusted third party. The cloud server uses an encrypted index ${\hat{I}}_{s}^{k}$, that is comprised of encrypted keywords. To compromise the privacy of the outsourced data, the cloud server either has to decipher the inverted index or deduce information from the evaluation of the encrypted search queries. In addition, the search queries are submitted in an encrypted format ($\alpha_{0\ldots l}^{\sigma_{sk}}$), and are evaluated by using a private matching protocol i.e., ($P\left(y_{0\ldots n}\in {\hat{I}}_{kw_{0\ldots n}}^{\omega_{u}}\right)=\Delta_{y_{0\ldots n}}$). Since, search queries are encrypted and the result of query evaluation is oblivious to the cloud server, the cloud server cannot learn any information about the keywords concealed in the search query.

In order to compromise the privacy of the outsourced data, the cloud server needs access to the secret key, *k*, which is shared by the repository owner. Once the cloud server has access to the secret key it can effortlessly decipher the keywords that comprises the inverted index. However, only authorized subscribers have access to the secret key as it is encrypted with their respective public key ($\omega_{u_{i}}^{k_{pub}}$). Thus, for a cloud server the computational complexity to compromise the privacy of the outsourced is equivalent to that of asymmetric encryption. However, even if the cloud server manages to gain access to the secret key it can only decipher the encrypted keywords that are associated with the outsourced data—so that confidentialitly of the outsourced data is preserved as it is encrypted with a symmetric encryption key, which is only disseminated to authorized subscribers. Since our proposed methodology deals with the encrypted data search, the topic of authorized data access is beyond its scope.

### Malicious Subscriber

The proposed methodology of encrypted data search not only realizes encrypted search in untrusted domain it also tackles the problem of unauthorized data search by malicious users. It ensures that unauthorized subscribers are not able to deduce any information about the encrypted outsourced data by simply learning the presence or absence of keywords. It does provide protection against conspired attacks by unauthorized subscribers and untrusted cloud server. Since, encrypted index is concealed with secret key that is only shared amongst authorized collaborating subscribers, malicious subscriber can not successfully evaluate their search query.

To search the encrypted data, the search criteria ($C_{kw_{0\ldots l}}$) is concealed with a secret key i.e., $E_{S}\left({\hat{C}}_{kw_{0\ldots l}},k\right)={\hat{C}}_{kw_{0\ldots l}}^{k}$. Once concealed it is then used to the model search query, which is comprised of the encrypted coefficients $\alpha_{0\ldots l}^{\sigma_{sk}}$, of polynomial $P(x\in {\hat{C}}_{kw_{0\ldots l}}^{k})$.

Since, only authorized subscribers have secret keys, search queries from unauthorized subscribers cannot be evaluated successfully. Also, unauthorized subscribers cannot intercept valid search query to modify the search criteria. This is because unauthorized subscriber does have valid secret key to model new or a part of valid search request. The concealed search criteria are only comparable with the encrypted index if the search criteria are also encrypted with the same key. Even if unauthorized subscribers collude with the cloud server, the execution of unauthorized search queries cannot assist them in learning any useful information. The search criterion encrypted with the arbitrary secret key is not compatible with the concealed inverted index, i.e., ${\hat{C}}_{kw_{0\ldots l}}^{k_{?}}\notin {\hat{I}}_{kw_{0\ldots n}}^{k}$. Thus, for unauthorized subscribers, it is computationally infeasible to deduce any information that can lead to the potential loss of data privacy.

## Conclusion and Future Directions

This paper addresses the problem of privacy-aware data search within the untrusted domain of a cloud service provider. It proposes an index-based privacy-aware data search methodology which can identify a semantic match between encrypted data and search criteria. Unlike the conventional methodology, the proposed privacy-aware data search leverages authorized subscribers to access relevant data by defining conjunctive search queries without relying on any trapdoors defined by the data owner. It realizes an oblivious data search, which ensures that the cloud service provider can only assist in the execution of encrypted search queries; however, the CSP can not learn or deduce confidential information from the execution of the search query, which can lead to the potential loss of data privacy. The security analysis demonstrated that, for malicious subscribers and untrusted cloud service providers, the proposed methodology always generates a randomized response that restrains them from learning of the presence or absence of a particular keyword in the outsourced encrypted data. Since the proposed methodology is an index-based data search, it does not have a requirement to encrypt the outsourced data with a particular encryption algorithm. The encryption of outsourced data with an arbitrary encryption algorithm does not affect the operation of the proposed methodology. The computational analysis shows that the proposed methodology exerts a reasonable computational load on authorized subscribers to model their encrypted search queries.

So far, the proposed methodology can only identify exact matches between the extended search criteria and the inverted index. In the future, we plan to include wildcard-enabled search queries, which can be used to match a substring while ensuring oblivious execution and privacy-awareness of search queries.
